# Rapid bone destruction caused by multidrug-resistant *Pseudomonas aeruginosa* septic arthritis: A case report

**DOI:** 10.1097/MD.0000000000039462

**Published:** 2024-09-06

**Authors:** Sung Cheol Yang, Yong In, Saad Mohammed AlShammari, Man Soo Kim

**Affiliations:** a Department of Orthopedic Surgery, Seoul St. Mary’s Hospital, College of Medicine, The Catholic University of Korea, Seoul, Republic of Korea; b Department of Orthopedic Surgery, King Abdulaziz Air Base Hospital, Ministry of Defense, Dhahran, Saudi Arabia.

**Keywords:** multidrug resistant, osteolysis, pseudomonas, septic arthritis

## Abstract

**Rationale:**

Infections due to multidrug-resistant (MDR) *Pseudomonas aeruginosa* are strongly associated with poor outcomes, including prolonged hospitalization and an increased risk of mortality. Antimicrobial options for the treatment of severe infections due to MDR *P aeruginosa* are quite limited, and treatment remains challenging.

**Patient concerns:**

A 65-year-old woman presented to our orthopedic clinic with a 3-month history of progressive pain and stiffness in her left knee. Her primary care provider administered a hyaluronic acid injection, which unfortunately resulted in worsening symptoms. Subsequent treatment included a 1-month course of intravenous gentamicin and ceftriaxone, which failed to alleviate her symptoms.

**Diagnosis:**

MDR *P aeruginosa* septic arthritis of the knee. The culture isolate was tested for susceptibility to multiple antibiotics. Magnetic resonance imaging evaluations were conducted, showing notable erosive and osteolytic changes around the joint surfaces that had progressed significantly.

**Interventions:**

The patient underwent arthroscopic irrigation and synovectomy. The treatment regimen included a combination of intravenous colistin and piperacillin/tazobactam administered over a 6-week period. Total knee arthroplasty was performed 6 months later without additional antibiotic treatment.

**Outcomes:**

Patient’s knee condition remained continuously stable without abnormal findings of inflammation. The patient’s knee range of motion increased 0 to 125 degrees, her pain almost disappeared, and she was able to maintain activities of daily life.

**Lessons:**

This case underscores the challenges of managing infections with MDR organisms in complex clinical scenarios, emphasizing the need for timely intervention and appropriate antibiotic therapy.

## 1. Introduction

*Pseudomonas aeruginosa*, a gram-negative bacterium (GNB), is pathogenic in immunocompromised individuals and resistant to several antibiotics.^[1]^ Although infections with *P aeruginosa* are relatively rare in healthy individuals, they can result in severe and rapidly progressive conditions when they do occur, particularly in compromised joints.^[1]^ The increasing prevalence of multidrug-resistant (MDR) strains of *P aeruginosa* poses significant treatment challenges, as these infections often necessitate complex, multifaceted therapeutic approaches, including both surgical intervention and prolonged courses of proper antibiotics.^[2]^ The gravity of these infections is heightened in older patients or those with underlying health conditions, where the immune response may be diminished, and the risk of complications is higher.^[3]^ In particular, MDR *P aeruginosa* has emerged as a formidable pathogen, capable of causing extensive joint damage and leading to rapid deterioration even in patients without a history of severe immunosuppression.^[4,5]^

Infections due to MDR *P aeruginosa* are strongly associated with poor outcomes, including prolonged hospitalization and an increased risk of mortality.^[6]^ While the risk of death from *P aeruginosa* infections is high, the mortality rate is more than doubled when caused by MDR strains.^[7]^ Inadequate initial antimicrobial therapy may contribute to the high mortality rate from MDR *P aeruginosa* infections.^[7,8]^ Antimicrobial options for the treatment of severe infections due to MDR *P aeruginosa* are quite limited, and treatment remains challenging.^[2,9]^

This case report presents a unique instance of an MDR *P aeruginosa* septic knee infection in an elderly patient without significant immunosuppressive history, emphasizing the rarity and severity of such cases. The purpose of this report is to elucidate the complexities involved in diagnosing and managing septic arthritis caused by resistant strains of *P aeruginosa*. By detailing the clinical course, therapeutic interventions, and outcomes, this report aims to contribute valuable insights to the medical literature, aiding healthcare professionals in better understanding and addressing these challenging infections.

## 2. Case presentation

### 2.1. Patient information

A 65-year-old woman presented to our orthopedic clinic with a 3-month history of progressive pain and stiffness in her left knee. Although she was a 65-year-old elderly patient, she did not have any chronic diseases, including diabetes or high blood pressure, and had no significant family or psychological history. Her medical history was notable for a fractured left patella 8 years prior, which was intermittently symptomatic with episodes of effusion. Initially, her primary care provider administered a hyaluronic acid injection, which unfortunately resulted in worsening symptoms. The pain in the knee worsened, and swelling and warmth also occurred. The initial therapy with gentamycin and ceftriaxone was administered at another hospital where the treatment was first performed. Aspiration was performed, but no bacteria were detected, so gentamycin and ceftriaxone were used for 1 month under the suspicion of gram-negative pathogens.^[10]^ It is well known that the elderly and injection therapy are closely related to gram-negative strain infection.^[4,11,12]^ As there was no improvement despite antibiotic treatment, the patient was transferred to our hospital for additional treatment.

### 2.2. Findings and diagnostic assessment

**Figure 1.**
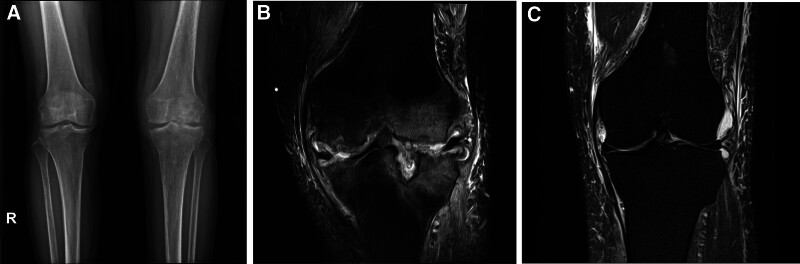
Radiography and magnetic resonance imaging revealed notable erosive and osteolytic changes around the joint surface (A and B), which had progressed rapidly since 3 mo prior (C). R = resistant.

Upon presentation, physical examination revealed painful restriction of motion from 10 to 90 degrees and mild joint effusion. Aspiration of the joint effusion was performed. The culture isolate was tested for susceptibility to multiple antibiotics (Table 1). MDR *P aeruginosa* was defined as follows: *P aeruginosa* was nonsusceptible to 1 or more agents in 3 or more antimicrobial categories (aminoglycosides, carbapenems, cephalosporins, fluoroquinolones, penicillins + b-lactamase inhibitors, monobactams, phosphonic acids, and polymyxins).^[4]^ MDR *P aeruginosa* was diagnosed, and there were no susceptibility results for polymyxins, including colistin. Concurrently, radiographic and magnetic resonance imaging evaluations were conducted, showing notable erosive and osteolytic changes around the joint surfaces that had progressed significantly compared to images taken 3 months prior (Fig. 1).

**Table 1:** MIC of *Pseudomonas aeruginosa* identified in synovial fluid culture.

Antibiotics	MIC	Susceptibility
Amikacin	4.0	S
Aztreonam	16.0	I
Cefepime	8.0	I
Ceftazidime	16.0	I
Ciprofloxacin	≥4.0	R
Gentamicin	4.0	S
Imipenem	≥16.0	R
Meropenem	≥16.0	R
Piperacillin	64.0	I
Piperacillin/tazobactam	≥128.0	R
Ticarcillin/clavulanic acid	≥128.0	R

I = intermediate, MIC = minimum inhibitory concentration, R = resistant, S = susceptible.

### 2.3. Therapeutic intervention

**Figure 2.**
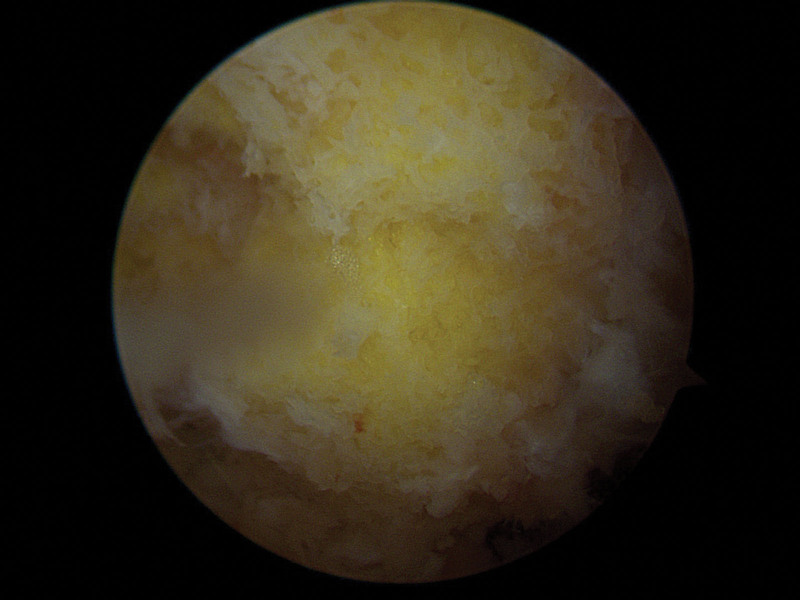
The patient underwent arthroscopic irrigation and synovectomy, during which diffuse synovitis and bony destruction across the joint surface were observed.

Given the severity of the infection and the resistance pattern of the pathogen, the patient underwent arthroscopic irrigation and synovectomy. During the procedure, diffuse synovitis and extensive bony destruction across the joint surface were evident (Fig. 2). Histological analysis of the tissues obtained during surgery showed acute chronic inflammation, necrosis, and abscess formation. These findings confirmed the diagnosis of septic arthritis due to MDR *P aeruginosa*. The treatment regimen included a combination of intravenous colistin and piperacillin/tazobactam administered over a 6-week period selected based on the sensitivity profile of the cultured bacteria and the judgment of an infectious disease specialist in collaboration with the infectious disease department. This combination is recommended in guidelines for managing severe infections caused by MDR *P aeruginosa*.^[3,5]^ The patient’s clinical response was monitored through inflammatory markers and repeat imaging alongside physical therapy to maintain as much joint function as possible.

### 2.4. Follow-up and clinical outcomes

Throughout the treatment period, the patient showed gradual improvement in symptoms. Follow-up visits over the subsequent weeks revealed resolution of the effusion and a significant reduction in pain. The range of motion in the knee improved modestly but was limited by the structural damage sustained. The patient was advised of the potential need for future surgical interventions, including possible joint preservation or replacement surgeries, depending on the long-term functionality of the knee and the progression of the joint damage.

**Figure 3.**
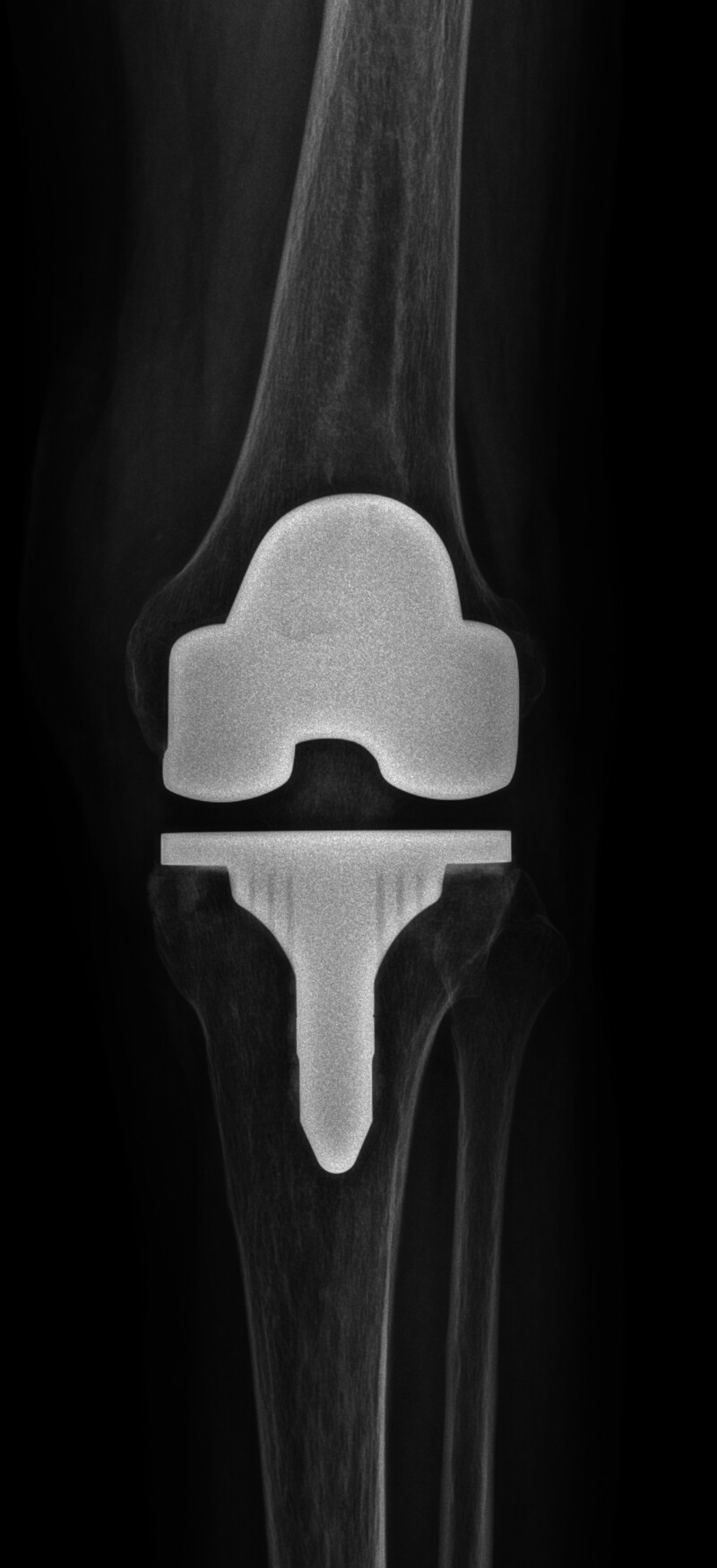
Total knee arthroplasty was performed after the patient’s septic arthritis was treated.

As the patient’s knee condition remained continuously stable without abnormal findings of inflammation, total knee arthroplasty was performed 6 months later without additional antibiotic treatment (Fig. 3). After surgery, the patient’s knee range of motion increased 0 to 125 degrees. The patient expressed significant relief from pain and improved ability to perform daily activities following the treatment. She reported satisfaction with the medical care received and was pleased with the overall outcome. In the case of polymyxin containing colistin, there is always a risk of nephrotoxicity. Renal impairment was defined as a decrease in glomerular filtration rate to 60 mL/min/1.73 m^2^ in previously normal renal function^[13]^ or a doubling of the baseline creatinine level or a 50% decrease in glomerular filtration rate in previously chronic renal dysfunction. Renal function tests were confirmed by repeat blood tests,^[14]^ and no renal impairment was found in the patient.

### 2.5. Ethical review and patient consent

The study was approved by the Institutional Review Board of Seoul St. Mary’s Hospital, an affiliate of the Catholic University of Korea (KC23ZISI0867). Written informed consent was obtained from the patient for publication of this case report.

## 3. Discussion

This case demonstrates that infection by antibiotic-resistant *P aeruginosa* can potentially have serious effects on joints. The rapid progression of joint destruction resulting from initial inappropriate antibiotics selection highlights the pathogen’s capacity to cause significant morbidity. *P aeruginosa* is notorious for its intrinsic resistance mechanisms, including efflux pumps, enzyme production, and biofilm formation, which contribute to its resilience against many common antibiotics.

Knee osteoarthritis is the most common form of degenerative arthritis,^[15–17]^ and septic arthritis accompanying knee osteoarthritis can sometimes be severe enough to destroy the joint.^[18]^ Septic arthritis is defined as an infection within the joint space caused by bacteria or other infectious agents.^[1]^ The most common risk factors in adult patients include older age, preexisting joint disease, recent joint surgery or injections, skin or soft tissue infections, intravenous drug abuse, indwelling catheters, immunosuppression, and diabetes.^[19]^ The pathogenesis of septic arthritis is generally associated with hematogenous seeding of the synovial membrane, which is highly vascular and susceptible to infection.^[20]^ *Staphylococcus aureus* is the most common cause of septic arthritis. Following *S aureus*, *Streptococcus* spp. is the next most commonly isolated bacteria in adult septic arthritis patients. Other less frequently encountered microorganisms include *Neisseria gonorrhoeae*, which is more common in young, sexually active adults, and various GNB such as *Escherichia coli*.^[20]^ *P aeruginosa* is a rare cause of septic arthritis but is a commensal organism commonly found on the skin, mucous membranes, and in the intestinal tract of humans.^[21]^ Infections caused by *P aeruginosa* primarily occur in patients with underlying medical conditions, and outbreaks of septic arthritis due to this organism typically occur in immunocompromised individuals, intravenous drug users, patients who have experienced significant trauma, or patients undergoing invasive procedures.^[21]^

For healthcare professionals, choosing effective antibiotics for MDR *P aeruginosa* infections is a challenge of global significance.^[2]^ The *P aeruginosa* identified in this case is an MDR *P aeruginosa* resistant to carbapenems, fluoroquinolones, and penicillins + β-lactamase inhibitors. The diagnosis of MDR *P aeruginosa* was based on antibiotic susceptibility testing, but the decision to use colistin-based combination antibiotics was made based on the clinical judgment and experience of the infectious disease specialist. In the treatment of MDR *P aeruginosa* septic arthritis, it is crucial to use appropriate antibiotics in conjunction with surgical intervention.^[22]^ Additionally, thorough debridement to remove the inflammatory tissue can enhance the effectiveness of antibiotics and should be carefully considered.^[22]^ The use of colistin-based combination antibiotics can be a viable option for treating MDR *P aeruginosa* septic arthritis.^[5,23–25]^

*P aeruginosa* poses significant challenges in clinical settings due to its resistance to many common antibiotics.^[26]^ This pathogen frequently exhibits resistance to β-lactams, often leaving polymyxins and aminoglycosides as the only effective treatments.^[26]^ Managing osteoarticular infections caused by MDR GNB, including *P aeruginosa*, introduces complex issues for healthcare providers, with no universally accepted treatment protocol currently established. The efficacy of β-lactams in these cases is particularly questionable.^[5]^ Recent studies suggest that combination therapy, particularly β-lactams paired with colistin, is more effective than monotherapy using either agent alone.^[5]^ Valour et al highlighted a series of bone and joint infections driven by MDR GNB, including 16 cases caused by *P aeruginosa*. They reported a 41% cure rate in orthopedic device–related infections, even after implant removal, through treatment with colistin alone.^[27]^ The findings by Ribera et al further corroborate the benefits of combination therapy, showing an enhanced cure rate of 71% when a β-lactam is used alongside colistin.^[5]^ These results underscore the potential of colistin in conjunction with β-lactams to effectively treat biofilm-associated infections, providing critical insights for optimizing therapeutic strategies against these formidable pathogens.^[28]^

Colistin-based antibiotics are known to be effective against MDR *P aeruginosa* and are considered a last resort for MDR gram-negative pathogens.^[5,23–25]^ Colistin, a polymyxin antibiotic, is often reserved for severe infections due to its potent activity against MDR organisms.^[3,5]^ Although our automated susceptibility panel did not include colistin, the infectious disease specialist initiated colistin-based treatment based on clinical judgment.^[5,23–25]^ Colistin is effective against the less active bacteria located in the deeper layers of biofilms, in contrast to most antibiotics that target the upper layers.^[5,23–25]^ Colistin rapidly alters the permeability of the cytoplasmic membrane in gram-negative pathogens, allowing other antibiotics to enter the cells.^[5,23–25]^ Despite the resistance results for piperacillin/tazobactam, it was used in combination with colistin due to the potential synergistic effect, where the combined use of 2 antibiotics results in a greater effect than when used alone.^[5,25]^ The use of combination therapies is advised for treating severe MDR infections according to current clinical protocols, due to their potential for enhanced bacterial eradication.^[2,3,5]^ Although these recommendations advocate for combination therapy, they are based on conditional evidence and lack robust support.^[2]^ Furthermore, existing guidelines fall short in clearly delineating criteria for diagnosing severe MDR infections.^[2]^ Given the increasing incidence of MDR *P aeruginosa*, it is necessary for hospitals to include antibiotic susceptibility testing for polymyxins, including colistin.

In our case, the patient presented with significant joint damage, including erosive and osteolytic changes, due to the destructive nature of the *P aeruginosa* infection and inadequate antibiotics selection. Significant joint damage was experienced prior to initiation of optimal management, including immediate surgical intervention and targeted antibiotic therapy. This case adds to the growing literature on the severe outcomes associated with resistant *P aeruginosa* infections and serves as a reminder of the challenges faced in treating these infections.^[2]^ It highlights the need for early recognition, appropriate antibiotic selection, and possibly the development of new therapeutic strategies to better manage such infections.

## 4. Conclusion

In conclusion, destructive knee joint infections caused by resistant pathogens such as *P aeruginosa* require a prompt and comprehensive treatment approach to minimize morbidity and preserve joint function. This case report contributes valuable insights into the management of such challenging infections, emphasizing the need for vigilant diagnosis, effective treatment strategies, and interdisciplinary collaboration to achieve favorable outcomes. Further research is warranted to explore novel treatments and strategies to combat antibiotic resistance and improve patient prognosis in similar cases.

## Author contributions

**Data curation:** Sung Cheol Yang, Saad Mohammed AlShammari, Man Soo Kim.

**Formal analysis:** Sung Cheol Yang, Man Soo Kim.

**Methodology:** Sung Cheol Yang, Man Soo Kim.

**Writing – original draft:** Sung Cheol Yang.

**Supervision:** Yong In, Man Soo Kim.

**Validation:** Yong In, Man Soo Kim.

**Writing – review & editing:** Yong In, Man Soo Kim.

**Conceptualization:** Man Soo Kim.

**Funding acquisition:** Man Soo Kim.

**Investigation:** Man Soo Kim.

**Visualization:** Man Soo Kim.
